# *Streptococcus suis* Infection in Hospitalized Patients, Nakhon Phanom Province, Thailand

**DOI:** 10.3201/eid2102.140961

**Published:** 2015-02

**Authors:** Prabda Praphasiri, Jocelynn T. Owusu, Somsak Thammathitiwat, Darunee Ditsungnoen, Pimpawan Boonmongkon, Ornuma Sangwichian, Kriengkrai Prasert, Sankhom Srihapanya, Kanlaya Sornwong, Anusak Kerdsin, Surang Dejsirilert, Henry C. Baggett, Sonja J. Olsen

**Affiliations:** Thailand Ministry of Public Health–US Centers for Disease Control and Prevention Collaboration, Nonthaburi, Thailand (P. Praphasiri, J.T. Owusu, S. Thammathitiwat, D. Ditsungnoen, O. Sangwichian, H.C. Baggett, S.J. Olsen);; Mahidol University, Nakhon Pathom, Thailand (P. Praphasiri, P. Boonmongkon);; Nakhon Phanom Provincial Health Office, Nakhon Phanom (K. Prasert, S. Srihapanya, K. Sornwong);; Thailand Ministry of Public Health, Nonthaburi (A. Kerdsin, S. Dejsirilert);; Centers for Disease Control and Prevention, Atlanta, Georgia, USA (H.C. Baggett, S.J. Olsen)

**Keywords:** Streptococcus suis, meningitis, sepsis, Thailand, bacteria, zoonoses

## Abstract

In Nakhon Phanom, Thailand, we identified 38 hospitalized patients with *Streptococcus suis* infection during 2006–2012. Deafness developed in 12 patients; none died. Thirty-five reported recent exposure to pigs/pork. Annual incidence was 0.1–2.2 cases/100,000 population (0.2–3.2 in persons >20 years of age). Clinicians should consider *S. suis* infection in areas where pig exposure is common.

*Streptococcus suis*, a zoonotic pathogen found primarily in pigs, can cause serious infection in humans. Most cases in human occur in Southeast Asia, where pig rearing is common (*1*). In a recent global review, Thailand had the second highest number of reported cases, accounting for 11% of all reported cases worldwide (*2*). In Thailand, the first 2 cases of *S. suis* in humans were reported in 1987 (*3*). From 1997 (when *S. suis* infection was first reportable) through 2010, a total of 692 cases were reported (0–207 per year); nearly half were from northern Thailand (*4*). The national annual crude incidence rate was 0–0.381 per 100,000 persons (Table 1). The objective of this study was to describe persons hospitalized with, and incidence of, *S. suis* infection in Nakhon Phanom during 2006–2012. 

**Table 1:** Incidence of *Streptococcus suis*, Nakhon Phanom Province, Thailand, 2006–2012

Year	Nakhon Phanom				Thailand		
	Population, all ages	Active surveillance			Population, all ages†	Passive surveillance	
		No. cases	Crude incidence*			No. cases‡	Crude incidence*
1997					55,747,667	1	0.002
1998					55,747,667	0	0.000
1999					55,747,667	1	0.002
2000					55,747,667	1	0.002
2001					56,305,980	3	0.005
2002					56,840,337	1	0.002
2003					57,345,943	1	0.002
2004					57,830,569	1	0.002
2005					58,319,021	10	0.017
2006	734,000	2	0.27		58,755,907	41	0.070
2007	738,184	1	0.14		59,199,510	90	0.152
2008	742,500	2	0.27		59,626,014	106	0.178
2009	746,655	3	0.40		60,037,264	229	0.381
2010	751,251	8	1.06		60,435,937	207	0.343
2011	754,931	5	0.66				
2012	758,388	17	2.24				

*Per 100,000 persons. Blank cells indicate that data were not reported. †1997–1999 is assumed to be the same as 2000. ‡In 2010, five cases were in persons from Nakhon Phanom; for all other years, 0 cases occurred.

## The Study

In 2003, the Thailand Ministry of Public Health and the US Centers for Disease Control and Prevention established hospital-based surveillance for community-acquired acute lower respiratory infections (ALRI) at all 12 acute-care hospitals in Nakhon Phanom Province (northeastern Thailand; population 761,623) (*5*). In 2005, surveillance was expanded to include bloodstream infections, supported by the addition of an automated blood culture system and improved microbiology capacity (*6*). Blood was collected for culture at clinician discretion but encouraged for all patients with ALRI and children <5 years of age who had sepsis. Incidence of pneumococcal bacteremia (all ages) and other bloodstream infections (children <5 years) was previously published (*6**,**7*). This work was considered public health surveillance and thus exempt from institutional review board review.

Blood put into a blood culture bottle was transported at 15–30°C within 24 hours to the provincial hospital laboratory and processed by using the BacT/ALERT 3D automated blood culture system (bioMérieux, Durham, NC, USA). To obtain at least 10 mL per adult patient, we divided specimens into 2 bottles (standard aerobic growth and enhanced growth of fastidious pathogens). Bottles that indicated positive growth were subcultured and processed by standard methods (*8*). All possible pathogens were confirmed at the National Institute of Health, Ministry of Public Health, by conventional biochemical tests (*9*). We serotyped *S. suis* isolates using PCR (*10*) and confirmed serotypes by coagglutination using rabbit antiserum (Statens Serum Institut, Copenhagen, Denmark). A case of *S. suis* was defined as illness in a person hospitalized in Nakhon Phanom who had blood culture–confirmed *S. suis* infection. Two physicians (K.P. and S.S.) reviewed the medical data retrospectively. Patients were interviewed by using a standard protocol (http://www.boe.moph.go.th/files/report/20100902_39823811.pdf). We calculated annual incidence using the estimated population as the denominator (http://www.nesdb.go.th/temp_social/pop.zip).

During 2006–2012, there were 56,983 blood cultures from 56,057 patients, an average of 8,008 patients per year (for comparison, in 2005, before microbiology enhancements, 2,340 patients had blood cultured at the provincial hospital). Median age of patients was 44 years (range 23–73 years). A pathogen was identified in 4,097 (7.2%) patients and *S. suis* in 38 (0.07%). Of the 38 *S. suis* cases, two occurred in 2006, one in 2007, two in 2008, three in 2009, eight in 2010, five in 2011, and 17 in 2012. Fifty-five percent of cases were identified during April–June (Figure). The annual crude incidence ranged from 0.1 to 2.2 cases per 100,000 population; incidence was highest in 2012 (Table 1). Of persons >20 years of age (all 38 *S. suis* patients), incidence was highest in 2012 (3.2 cases/100,000 population [range 0.2–3.2/100,000]).

**Figure:**
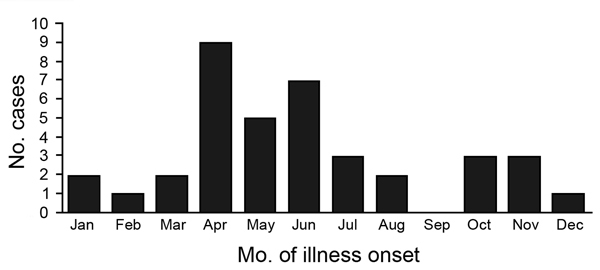
Cases of *Streptococcus suis* infection, by month of illness onset, Nakhon Phanom Province, Thailand, 2006–2012.

Within 24 hours after hospital admission, all patients were treated with ceftriaxone. In 12 (32%) patients, permanant deafness developed; all had reported hearing loss at admission. Thirty-five (92%) patients had exposure to pigs or pork in the 7 days before illness onset: 10 (26%), all women, reported preparing pork with their bare hands for consumption and eating undercooked pork, 12 (32%) reported eating both undercooked/uncooked pork and clotted pig blood, and 13 (34%) reported slaughtering pigs for their own consumption. Thirteen patients who reported slaughtering pigs also ate pork but stated that they could not recall how the meat was prepared because of having also consumed alcohol. Seven (18%) patients had acquired pigs in poor health from commercial farms at reduced prices or no cost. Patients resided in 18 (19%) of the 96 subdistricts within Nakhon Phanom, and 10 (26%) patients resided in the same subdistrict. Two clusters of cases occurred in 2012, in which 2 and 3 persons ate raw pork and drank alcohol together. All patients reported no prior knowledge of *S.* suis infection, its symptoms, or ways to prevent infection.

Of the 24 patients with meningitis, 21 (88%) had leukocytosis, 4 (17%) had thrombocytopenia, and 2 (8%) had thrombocytosis. Six of the 24 patients with meningitis had a cerebrospinal fluid (CSF) culture; 1 was positive for *S. suis*. Of the 10 patients with septicemia, 5 had leukocytosis, 1 had leukopenia, 3 had normal leukocyte counts; for 1, leukocyte count was unavailable. Thirty (79%) isolates were initially reported as *Streptococcus* group D nonenterococci by the hospital laboratory; the remaining 8 were reported as other streptococcal groups or species (Table 2). The National Institute of Health reference laboratory identified *S. suis* in 38 patients; all isolates were serotype 2 (PCR and coagglutination results were all concordant). All isolates tested for antimicrobial resistance by disk diffusion (Kirby-Bauer) were susceptible to penicillin (37 isolates) and ceftriaxone (11 isolates). Time from patient blood collection to final pathogen report to the clinician was 30–45 days.

**Table 2:** Characteristics of 38 patients for whom blood culture confirmed *Streptococcus* *suis* infection, Nakhon Phanom Province, Thailand, 2006–2012

**Characteristic**	**Result of analysis or culture**
**Male sex, no. (%)**	28 (74)
**Median age, y (range)**	50 (23–73)
Heavy alcohol use, no. (%)*	20 (53)
**Current smoker, i.e., smoked daily, no. (%)**	21 (55)
**Underlying chronic disease, no. (%)**	12 (32)
Hypertension	4 (33)
Diabetes	3 (25)
Alcoholism†	3 (25)
Heart disease	1 (0.8)
Gout	1 (0.8)
**Occupation: farmer, no. (%)‡**	33 (87)
**Consumption of/contact with pig or pork product, no. (%)**	35 (92)
**Days from pork/prok contact to illness onset, median (range)**	2 (1–7)
**Days from illness onset to hospital admission, median (range)**	2 (0–5)
**Clinical features**	
Meningitis, no. (%)	24 (63)
Septicemia, no. (%)	10 (26)
Arthritis, no. (%)	4 (11)
**Laboratory investigation**	
** Complete blood count, n = 37**	
** Leukocytosis, >10,000 cells/**μL, no. (%)	28 (76)
** Leukopenia,<5,000 cells/**μL, no. (%)	1 (3)
** Thrombocytosis, >450,000 cells/**μL, no. (%)	2 (5)
** Thrombocytopenia, <150,000 cells/**μL, no. (%)	7 (19)
** CSF examination,**§ n = 12	
** Protein, mean ± SD**, %	580 ± 421
** Glucose, mean ± SD**, mg/μL	23 ± 19
** Leukocyte count, mean ± SD**, cells/μL	2,210 ± 1,580
** Polymorphonuclear neutrophils, mean ± SD, %**	54 ± 28
** Blood culture result **reported by hospital laboratory¶	
***Streptococcus* group D, non-enterococci, no. (%)**	30 (79)
***Streptococcus* group D, no. (%)**	2 (5)
***Streptococcus pyogenes*, no. (%)**	1 (3)
** Group A **β-hemolytic *Streptococcus*, no. (%)	1 (3)
** β-Hemolytic *Streptococcus*, no. (%)**	1 (3)
***Streptococcus* not group A,B,D, no. (%)**	1 (3)
***Enterococcus* spp., no. (%)**	1 (3)
***Streptococcus s*pp., no. (%)**	1 (3)
CSF culture results reported by hospital laboratory, n = 6	
***Streptococcus* group D, nonenterococci, no. (%)**	5 (83)
***Streptococcus suis*, no. (%)**	1 (17)
**Days hospitalized, median (range)#**	7 (3–19)
**Death, no. (%)**	0
Permanent deafness, no. (%)**	12 (32)

*Defined as self-reported drinking of >2 alcoholic beverages/day or >14 drinks/week for men and >1 alcoholic beverage/day or >7 drinks/week for women. †In 1, cirrhosis also was diagnosed. ‡Three raised livestock, and 30 harvested crops (rice). §Reference values: protein, 15–45 mg/μL; glucose, 50–80 mg/μL; leukocytes, 0–5 cells/μL; polymorphonuclear neutrophils, 0%. ¶All 38 blood culture results were confirmed to be S. suis. #Excludes 2 patients who experienced septic shock and were transferred to the regional hospital (in another province) on the first day of admission. **Ten patients had bilateral deafness, of whom 2 with deafness reported chronic ataxia; all 12 had meningitis.

## Conclusions

*S. suis* infection is common in northern Thailand. Here we report laboratory-confirmed cases and incidence in Nakhon Phanom, a northeastern province. Few other studies have reported incidence. The Netherlands reported the most *S. suis* infections in the West (*2*) with an estimated annual incidence of *S. suis* infection of 0.002 cases per 100,000 persons (*11*), and the incidence in northern Thailand was 6.2 cases per 100,000 persons (*12*). Active surveillance suggests that *S. suis* infection might be more common in this region than previously realized (e.g., in 2010, the incidence in Nakhon Phanom was 1.6-fold higher in active than passive reporting; for other years it was greater). During 2006–2012, a total of 45% (17/38) of *S. suis* infection were detected in 2012, including 2 clusters.

Although we did not have a control group with which to compare exposures, our findings are consistent with studies performed in northern Thailand that highlight pork/pig exposure, combined with alcohol use, as a risk factor (*13*). Unlike in cases reported in other studies (*12*), no patients reported here died. Patients were treated promptly with ceftriaxone on the first day of admission, which is standard empiric management of suspected sepsis or meningitis in these hospitals. Permanent hearing loss was common, and deafness is usually permanent when it occurs before treatment (*14*).

Our data have several limitations. Blood cultures were performed at clinician discretion and not necessarily for all patients with possible sepsis or meningitis, possibly resulting in missed cases or biasing our study toward the more clinically apparent or severe cases. Blood volume might have been too low for adequate pathogen yield. Only 6 patients had CSF cultures, and most blood and CSF cultures occurred after start of antimicrobial therapy. Therefore, meningitis patients with negative blood cultures might have been missed. Furthermore, because comprehensive examinations were not performed on patients after discharge, neurologic or cognitivie sequalae might have been missed.

Because most hospital laboratories in Thailand are not able to confirm *S. suis*, the infection might be misdiagnosed (*14*). Clinicians in high-risk areas, or who see patients with recent travel to high-risk areas, should have a low index of suspicion for *S. suis* infection among patients presenting with meningitis or sepsis and recent pig/pork exposure (*15*). Improving the capacity of local laboratories to identify *S. suis* will aid clinical management and facilitate outbreak detection and response. Rapid identification enables faster epidemiologic investigation and swift initiation of control measures (*2*).
